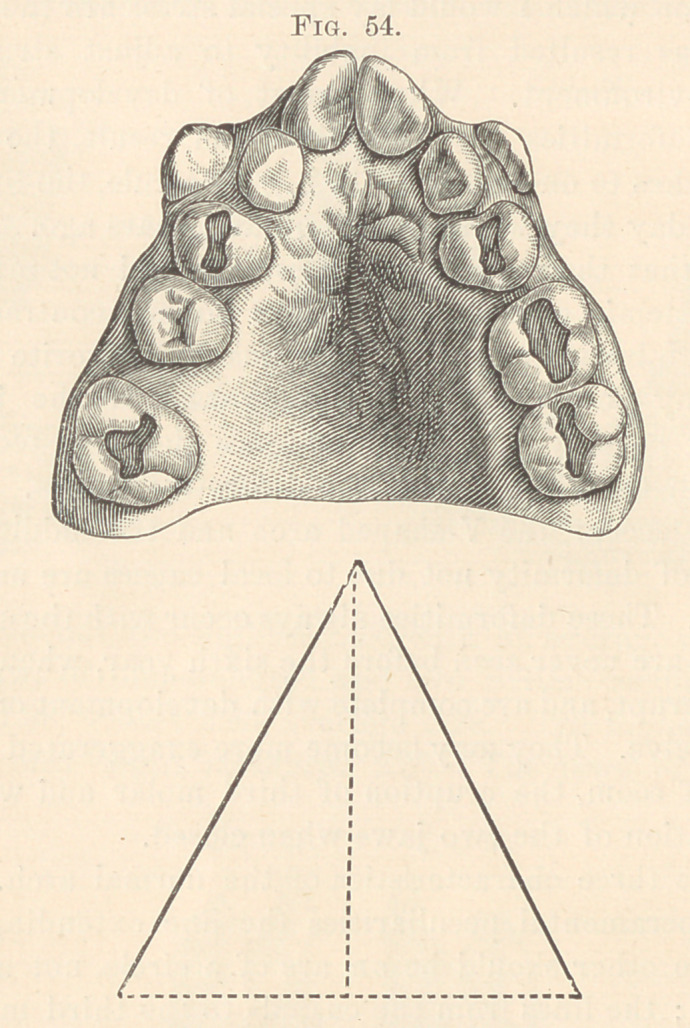# The Degenerate Jaws and Teeth

**Published:** 1897-03

**Authors:** Eugene S. Talbot


### International Dental Journal.

Vol. XVIII. | March, 1897. | No. 3.

### Original Communications.[1]

### THE DEGENERATE JAWS AND TEETH.[2]

BY EUGENE S. TALBOT, M.D., D.D.S.[3]

(Continued from page 85.)

Crescent-shaped bitubercular, tritubercular, as well as all deformed teeth tend to the cone shape. The malformation of these teeth results from precongenital trophic change in dentine development. It consists in dwarfing and notching the cutting and grinding edges of the second set of teeth, a familiar example of which is seen in the so-called Hutchinson teeth, usually referred to a syphilitic etiology. Hutchinson's position has, however, been more strongly stated than his words justify, since he admits that in at least one-tenth the cases luetic etiology could be excluded.[4]

Lues only plays the part of a diathetic state profoundly affecting the maternal constitution at the time of dentine development. While these teeth may be due to secondary result of lues, they do not demonstrate luetic heredity.

---

[1] The editor and publishers are not responsible for the views of authors of papers published in this department, nor for any claim to novelty, or otherwise, that may be made by them. No papers will be received for this department that have appeared in any other journal published in the country.

[2] Read in the Section on Neurology and Medical Jurisprudence at the Forty-seventh Annual Meeting of the American Medical Association, held at Atlanta, Ga., May 5 to 8, 1896. Reprinted from the Journal of the American Medical Association by special request.—[Ed.]

[3] Fellow of Chicago Academy of Medicine.

[4] American System of Dentistry.

In Fig. 31 are seen the teeth of an individual affected with constitutional disease, and by referring to Fig. 15 we shall see that

FIG. 31.

the defective lines represent the respective ages of two and a half, four, and five years. The degree of pitting will depend, as a rule, upon the severity of the constitutional disorder. In the case just cited, however, although nutrition was but slightly disordered, each tooth shows a tendency to conate. Not infrequently are cavities extended completely through the tooth. The cusps of the (permanent) first molars calcifying at the first year are usually attacked

FIG. 32.

also and arrested in development, producing the cone shape. These data, together with dates of eruption of the temporary and permanent teeth, furnish an absolute basis for calculation as to excessive or arrested development of tissue. Fig. 32 shows a very degenerate jaw with cone-shaped malformed bicuspids. The right lateral is missing, the cuspids are erupting in the vault, and the dental arch is assuming a V-shape. The jaw as a whole shows marked arrest in development. Fig. 33 shows "Hutchinson" teeth. Were the first molars visible they would present marked contraction of the

FIG. 33.

outer surface with a malformed centre. Referring again to Fig. 15 we observe that trophic changes affected the system at the age of birth. The outer surface exhibits a tendency to take the cone shape. Figs. 34, 35, 36, 37, and the molars in Fig. 30 exhibit mal-

FIG. 34. FIG. 35. FIG. 36. FIG. 37.

formations, assume the cone shape, and the centre frequently associated with this type of teeth. The coincidence in form between "Hutchinson" and malformed teeth and those of the chameleon demonstrates that tropho-neurotic change produces atavistic teeth. Fig. 38 illustrates the tendency of human bicuspids (when there is no antagonism) to rotate one-fourth round, thus again demonstrating the atavistic tendency towards the teeth of the chameleon. Fig. 39 exhibits extreme atavism; all teeth anterior to the molars are cone-shaped. The third molars are missing and would probably never erupt. In Fig. 40 appears more marked atavism. The upper and lower anterior are both cone-shaped and the superior first bicuspid exhibits a tendency thereto. The right superior second bicuspid, second and third molars, the right inferior first and second bicuspids, second and third molars are missing. The same condition probably exists on the left side. The space in the upper jaw is due to the insufficient width of the teeth. Alternation of teeth in the upper and lower jaws is a reptilian feature. Fig. 30 furnishes an excellent illustration of the principles hereinbefore advanced.

FIG. 38.

In degenerate jaws the influence of the factors of the differentiation theory are also demonstrated. Every tooth in the jaw at

FIG. 39.

one point or another may display rudimentary cusps. On the incisors they are always to be found on the lingual surface.

FIG. 40.

Fig. 41 illustrates the centrals with two rudimentary cusps, the laterals with one and the cuspids with one also. Fig. 42 represents cusps upon the lingual surfaces of the molars. The cuspids are not unlike the lower bicuspids with a rudimentary lingual cusp.

Thompson remarks that there is a gradation from central incisors towards the bicuspids in evolution. This grading of form is not observed as we pass from the cuspid to the bicuspid in man. But we must remember that the cuspid often presents a cingulum

Fig. 41.

on the lingual face that inclines it towards the bicuspid forms in lower mammals, like the mole, and that the first premolar or

Fig. 42.

bicuspid is then more caniniform, the inner tubercle being much reduced. This inner tubercle is very variable and erratic as to its position. It appears as far front as the centrals and is often present on the lingual face of the laterals of man. The lingual tubercle is very constant on the first bicuspid of man and is well developed as the buccal. But in some lower forms, as in the lemurs, it is quite deficient. It attains the highest development only in the anthropoids and man. Considering these stages of development, the grading from the cuspid to the bicuspid forms was more gradual in the earlier species than in the later, where the individual teeth have taken on special development.[1]

Fig. 43.

I have the skull of a degenerate girl who died from tuberculosis at thirteen years. Among other stigmata is a cusp on the external surface of a right inferior cuspid. This is a decidedly strong point in favor of the differentiation theory. Another strong point in favor of this theory is shown in Fig. 43, where every tooth is present and a most remarkable display of cusps occurs. The cusps upon the cutting and grinding edges are not obliterated. Commencing with the left superior central incisor three cusps are present with a rudimentary palatine cusp. The laterals also show three cusps, while the cuspid has two very distinct. The first and second bicuspids have tubercular cusps, they being in line. The buccal cusps upon the molars two to three and are still in position. The pala-

---

[1] Dental Cosmos, May, 1894.

tine cusps are worn away. The same is the case upon the opposite side except that the cuspid has cusps that have fused together, leaving a small projection upon the mesial side and a rudimentary palatine cusp. The cusp upon the third molar is lost. In another case (Fig. 25) the primitive cone teeth are seen trying to shape themselves into incisors. The lateral incisors, cuspids, and bicuspids are still cone-shaped. The first permanent molar is fairly formed, while the second molars are still in a primitive condition. Thus the points made by Osborn are nicely demonstrated in the two last illustrations,—namely, the triangular-shaped crowns and the levelling of cusps.

There is abundant evidence to show that degenerate teeth unite in twos, threes, fours, and fives, as indicated in the concrescent theory. These single cone-shaped teeth grow together and form bicuspids and molars. The germs of any two normal teeth may intermingle and unite; not only are the crowns found united with separate roots, but crowns and roots are united throughout.

Figs. 44 and 45 show two superior central and lateral incisors joined together throughout the entire length of crown and root;

Fig. 44.

Fig. 45.

Fig. 46.

Fig. 47.

Fig. 48.

Fig. 46, two lower incisors are united throughout; Fig. 47 shows a cuspid with two roots; Dr. George T. Carpenter, of Chicago, has a right superior second bicuspid with three well-formed roots; Fig. 48 illustrates two bicuspids united at the crowns; Fig. 49 shows two molars perfectly united; Fig. 50 illustrates central and lateral incisors of the permanent set perfectly united; Fig. 51 shows two molars united; Fig. 52 a molar and supernumerary united, the supernumerary taking the cone shape with deformed centre. Fig. 53 shows three malformed teeth, each conated and completely united.

It is not uncommon to find three molars united together, as for instance the second, third, and supernumerary molar. Dr. C. V. Rosser, Atlanta, Georgia, has two small molars and a supernumerary cuspid perfectly united from crown to root, and these three further united to the roots of a well-formed molar. Thus we see the concrescence theory is fully established.

That human jaws, like the human ears, are degenerating is a matter susceptible of demonstration by actual measurements. Mummery examined the skulls of two hundred Britons and Roman soldiers in Hythe church, Kent, England. He found the narrowest

Fig. 49.

Fig. 51.

Fig. 50.

width 2.12 inches, the highest 2.62, with an average of 2.50. The width of jaws of four hundred and two British soldiers to-day is: narrowest 1.88, widest 2.63, average 2.28. The highest width was very rare, only eight measured 2.50. The jaws of the mound-

Fig. 52.

Fig. 53.

builders compared with the existing cliff-dwellers show similar results: the average width is about 2.50 inches. This is also true of nearly pure negro races. Measurements of normal jaws of eight hundred and fifty-five Italians of central Italy were, narrowest 1.88, widest 2.63, average 2.17. Measurements of normal jaws of four thousand nine hundred and thirty-five Americans gave the following results: narrowest 1.75, widest, only one case, 2.56, average 2.13. If in the highest type of physical man the width of the upper jaw from the outer surfaces of the first permanent molars near the gum margin was originally 2.50 inches in diameter, the jaws of people now living in the same locality are from 0.25 to 0.33 inch smaller. Although the jaw has been growing smaller, since there are no breaks or deformities in the contour of the dental arch, this must be regarded simply as an adaptation to environment and not degeneracy in the proper sense of the term. The degeneracies of the jaws on which I would lay special stress are those in which deformity has resulted from inability to adjust structure to a changing environment. When arrest of development so takes place that deformities of the dental arch result, the jaws vary from two inches to one inch in width. As a rule, the teeth are the same size to-day they were three thousand years ago. This is due to the fact that their growth is antenatal and not influenced by postnatal systemic changes. The jaws do not contract as a result of mouth-breathing, that erroneous but favorite hypothesis with so many dentists and laryngologists. If the jaw can be arrested and be smaller in circumference than the teeth, a break takes place in the dental arch and deformity results. Two types of deformity occcur, the V-shaped arch and the saddle-arch. All other types of deformity not due to local causes are modifications of these two. These deformities always occur with the second teeth only. They are never seen before the sixth year, when the second set begin to erupt, and are complete with development of the second molars at twelve. They may become more exaggerated later in life from want of room, the eruption of third molar and want of harmony in relation of the two jaws when closed.

There are three characteristics of the normal arch. Independent of temperamental peculiarities the line extending from one cuspid to the other should be an arc of a circle, not an angle or straight line; the lines from the cuspids to the third molar should be straight, curving neither in nor out, the sides not approximating parallel lines. Absolute bilateral uniformity is not implied in this, as the two sides of the human jaw are rarely if ever wholly alike. A uniform arch necessitates a uniformity of development between the arch of the maxilla and the arch of the teeth and a correct position of the individual teeth in their relation to each other. When there is inharmony of development between the jaw and the teeth, as may happen when one parent has a small maxilla with correspondingly small teeth, and the other a large one with correspondingly large teeth, if the child inherits the jaw of one and the teeth of the other irregularities must follow. Such difference in diameter between the arch of the maxilla and that of the crowns of the teeth is a constitutional cause of irregularity. Whenever there is a difference between these diameters the line formed by the teeth must either fall outside or within the arch of the maxilla and irregularities of arrangements result. The primary divisions of irregularities are the V-shaped and saddle-shaped arches. We have the V-shaped variety (Fig. 54)[1] (one of the typical forms), where

Fig. 54.

the apex of a triangle is formed by the incisors, the base of the triangle being a line connecting the two first molars. If, because of premature or tardy extraction, the first molar moves forward or the coincidence of the arch of the maxilla and the arch of the crowns of the teeth in trying to accommodate itself to the lesser arch of the maxilla, becomes a broken line forming an angle at the incisors. This angle results from two causes, the thinness of the process at this point and the diminutition of resistance which must follow.

[1] While the general outlines of the jaw and teeth are the same, in no two cases are they exactly alike. The cuts, therefore, are not drawn from actual cases, but are ideal diagrams of typical cases.

(To be continued.)

## Figures and Tables

**Fig. 31. f1:**
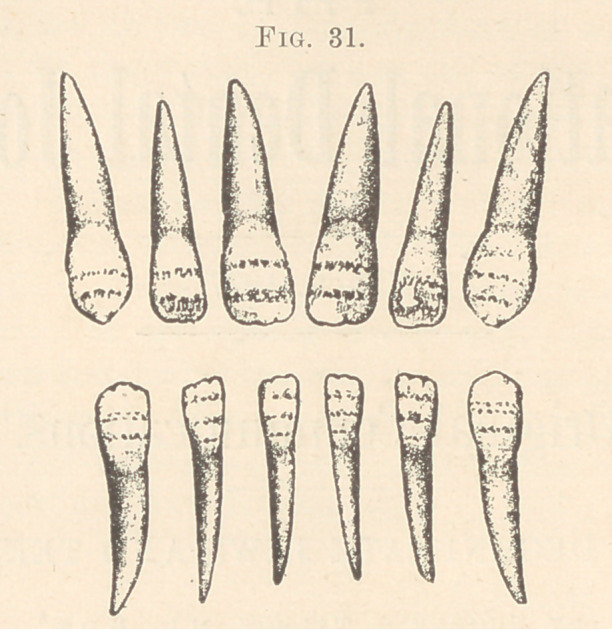


**Fig. 32. f2:**
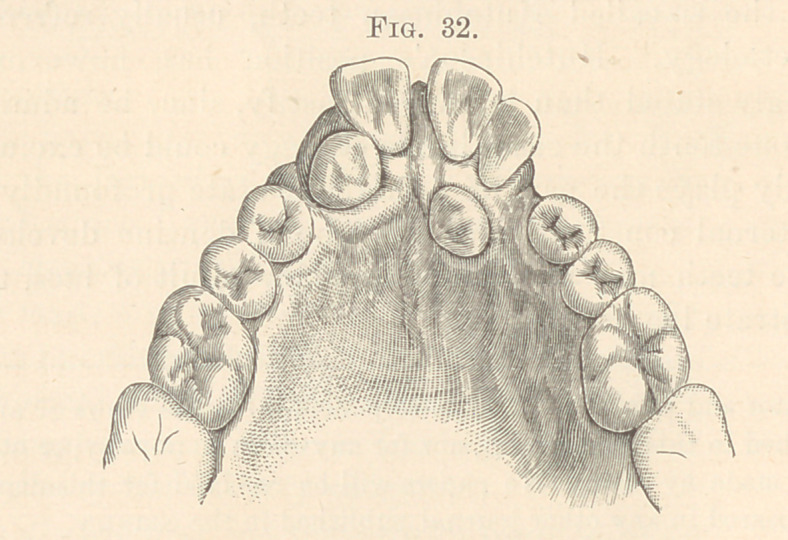


**Fig. 33. f3:**
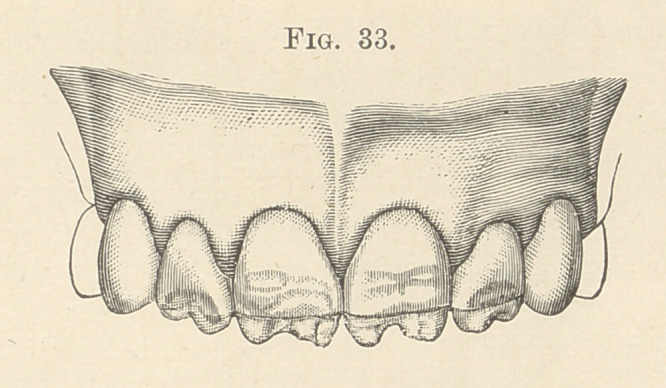


**Fig. 34. f4:**
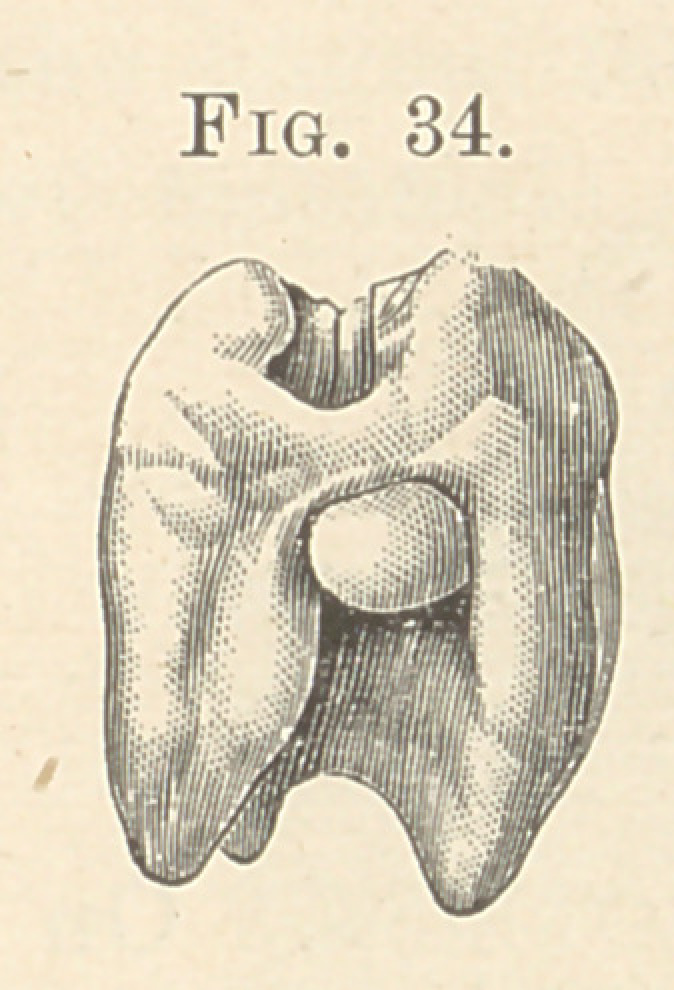


**Fig. 35. f5:**
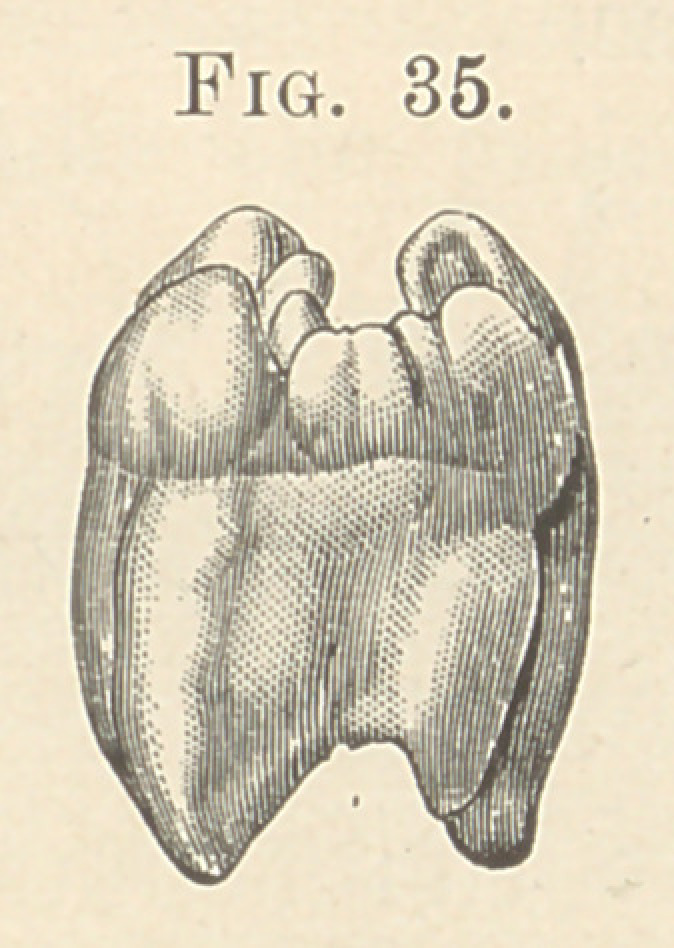


**Fig. 36. f6:**
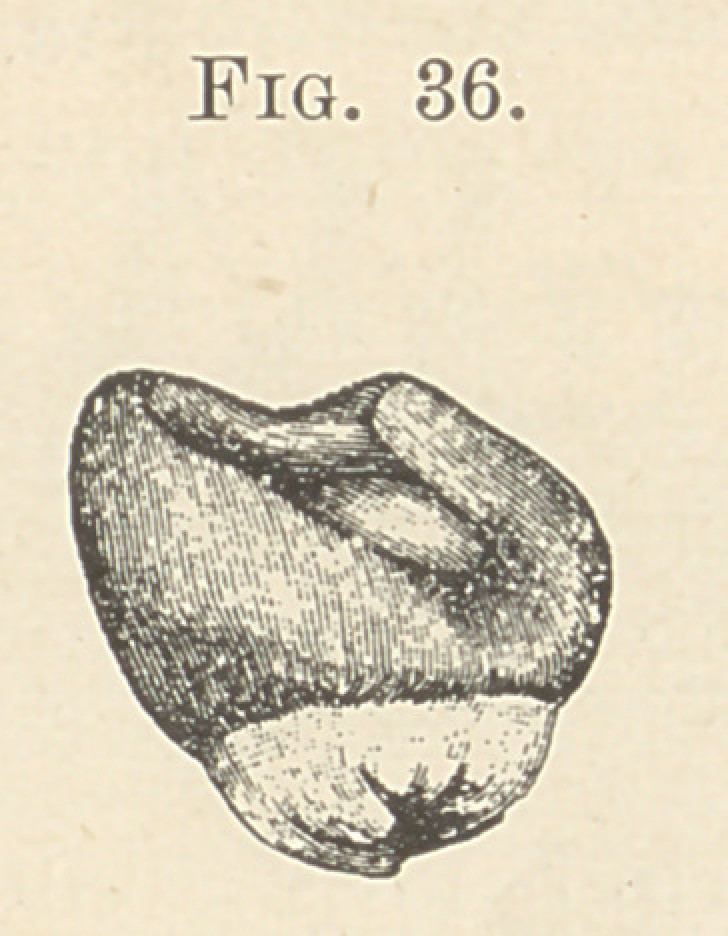


**Fig. 37. f7:**
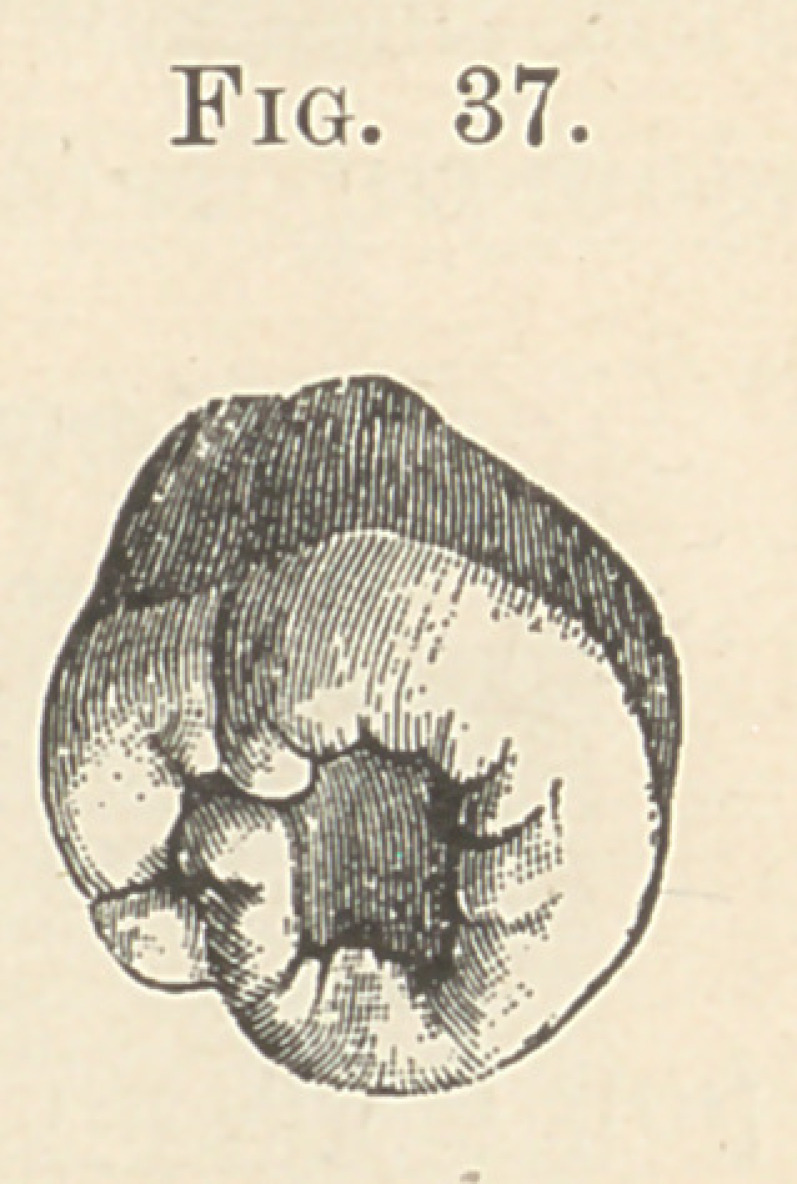


**Fig. 38. f8:**
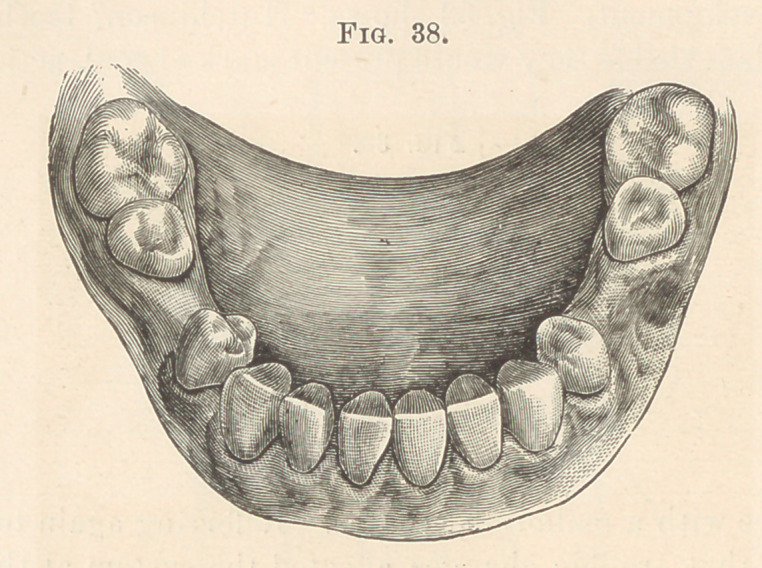


**Fig. 39. f9:**
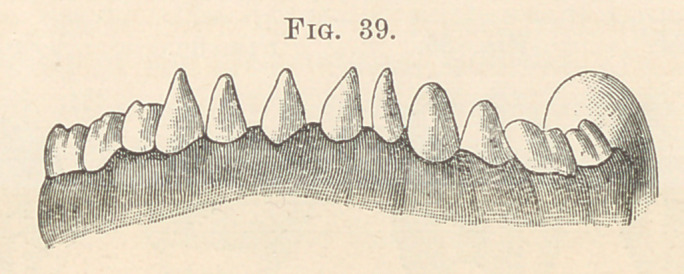


**Fig. 40. f10:**
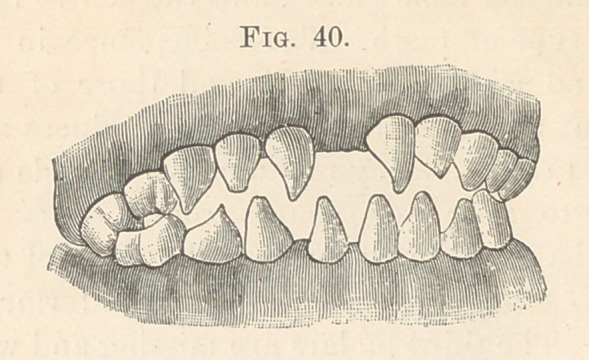


**Fig. 41. f11:**
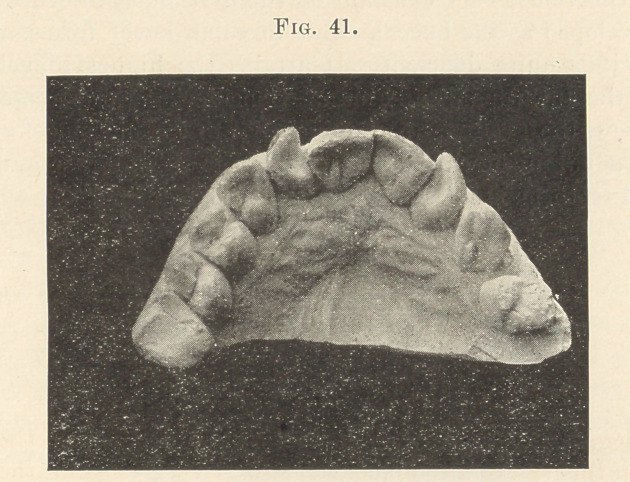


**Fig. 42. f12:**
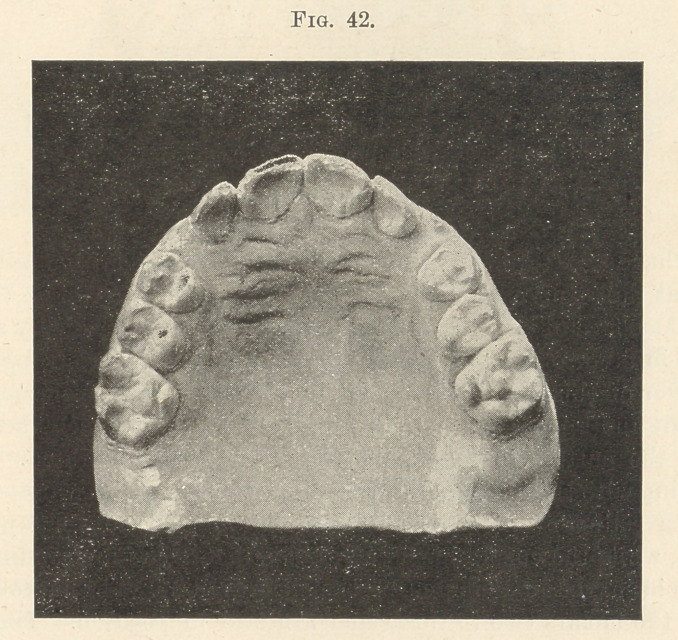


**Fig. 43. f13:**
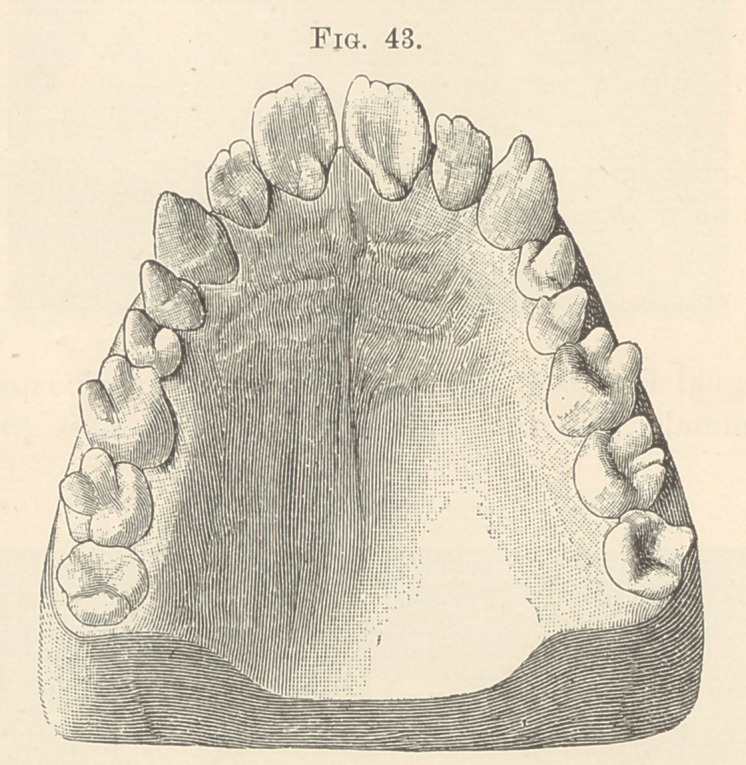


**Fig. 44. f14:**
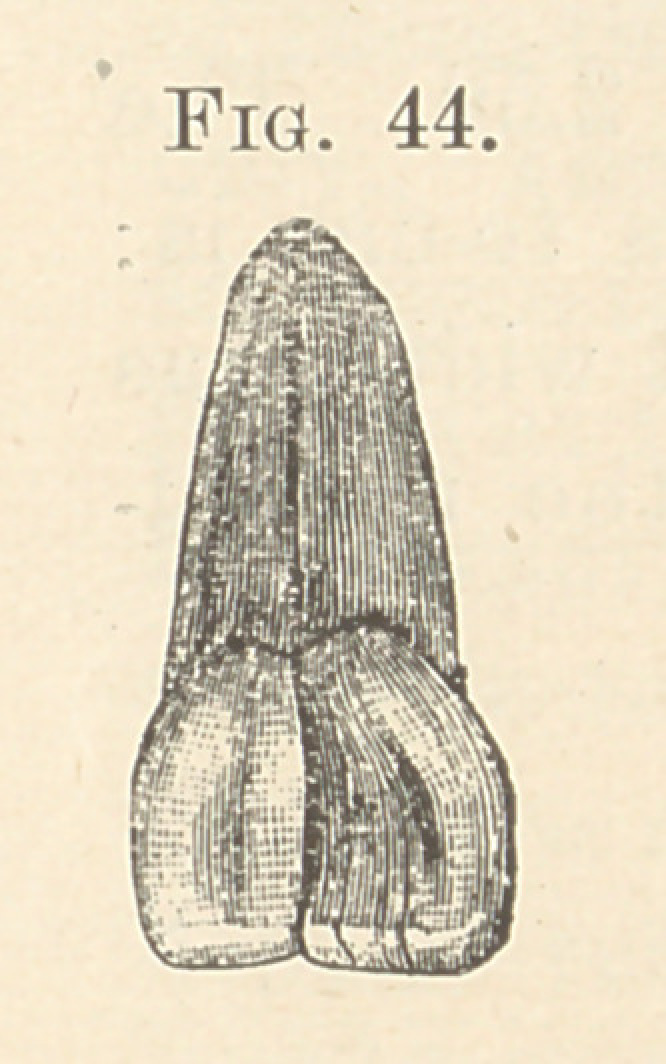


**Fig. 45. f15:**
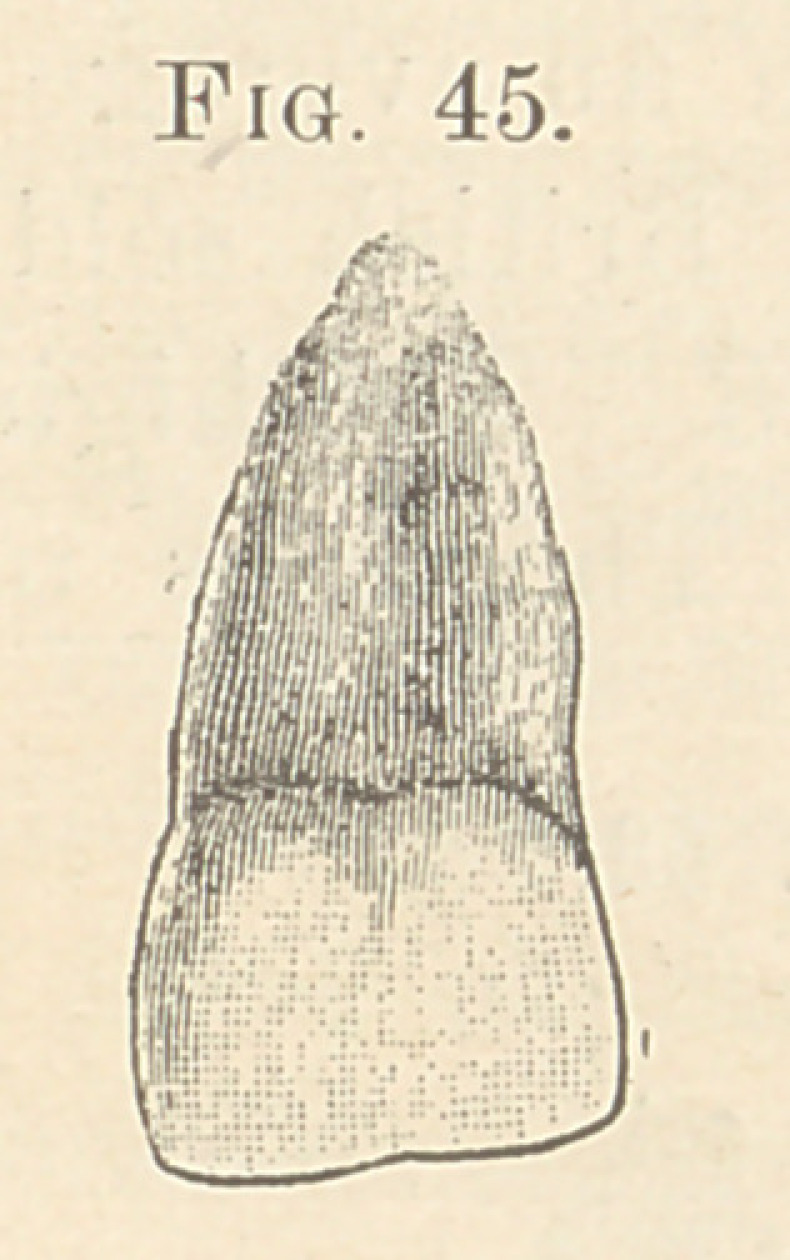


**Fig. 46. f16:**
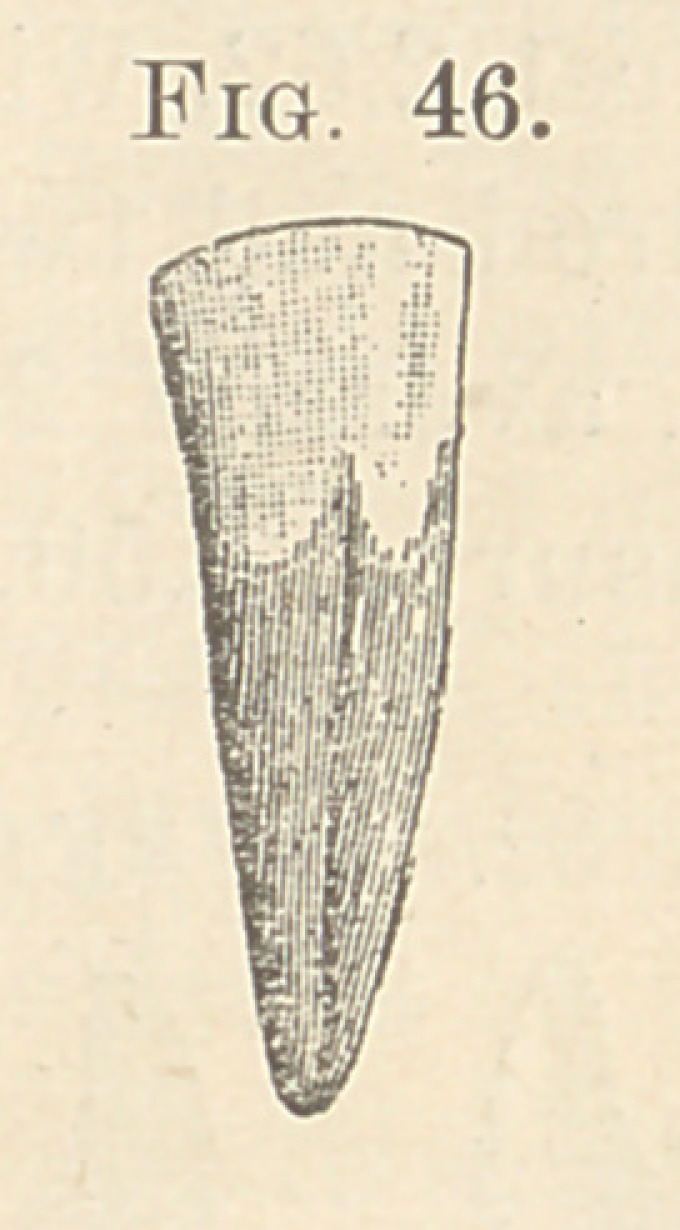


**Fig. 47. f17:**
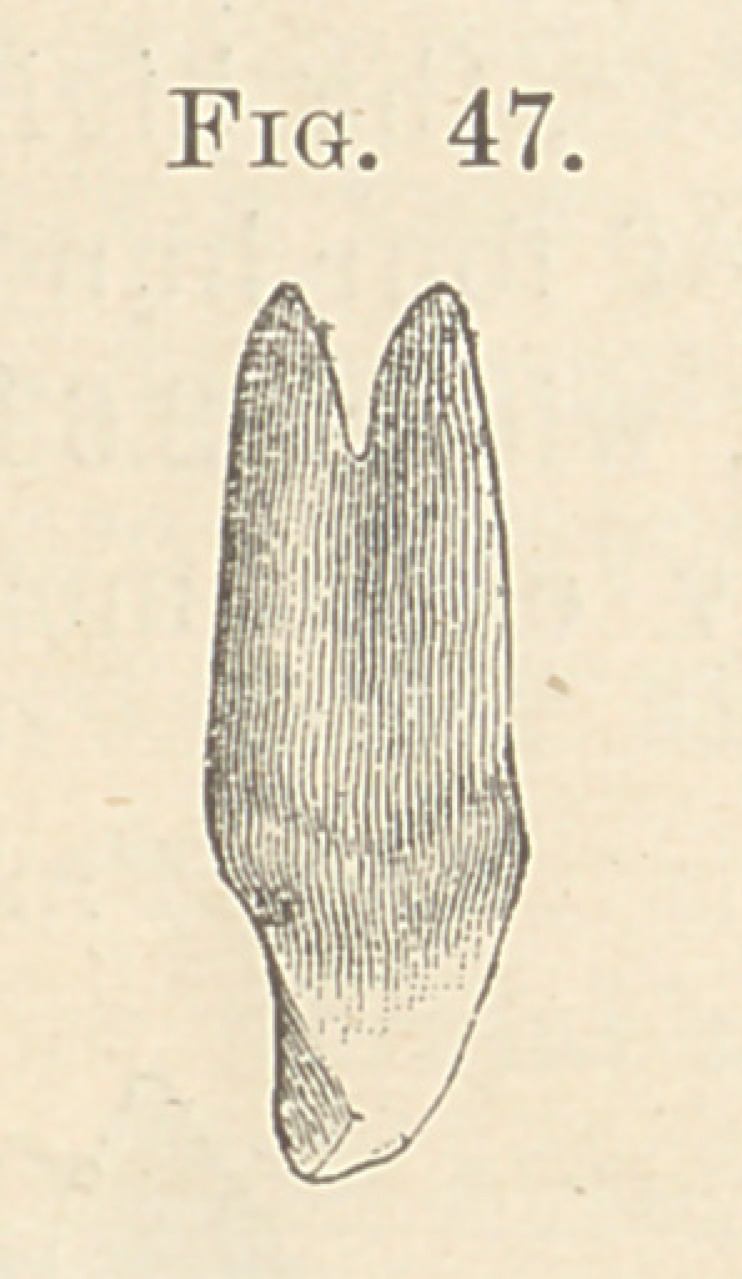


**Fig. 48. f18:**
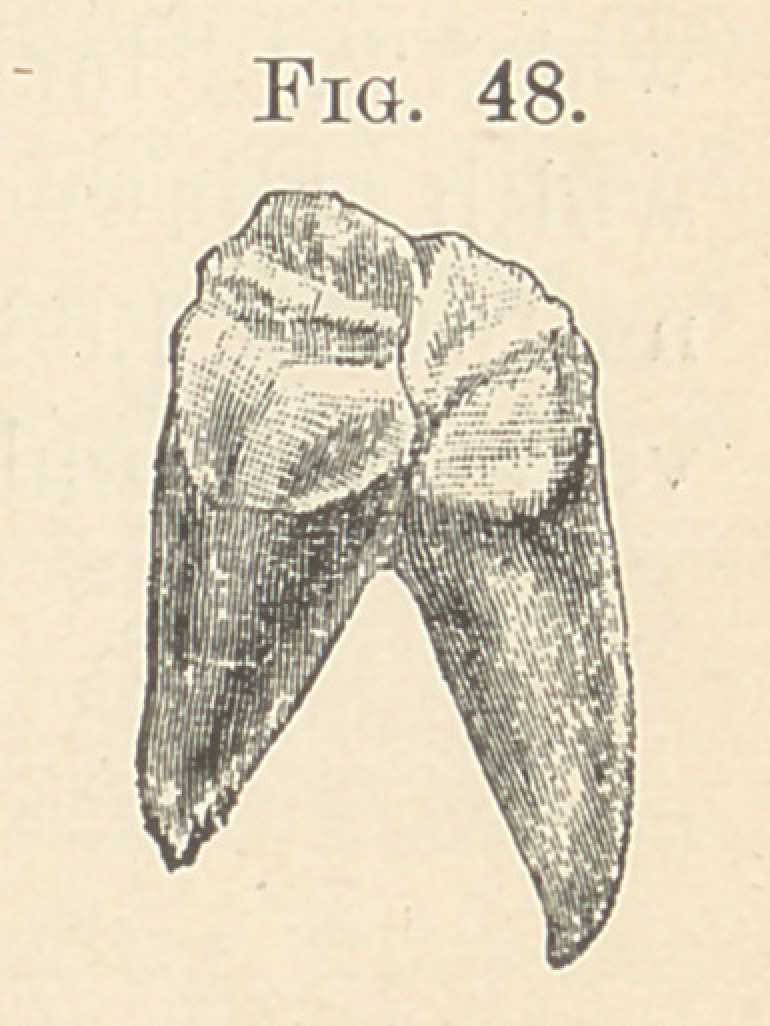


**Fig. 49. f19:**
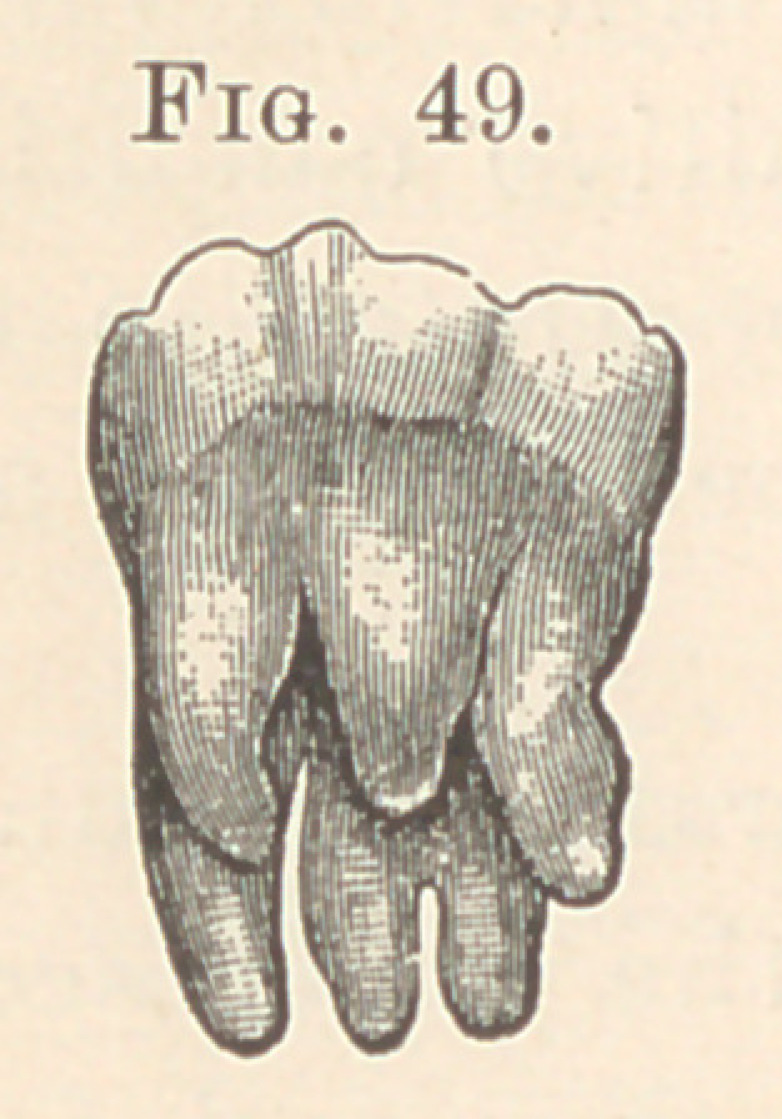


**Fig. 50. f20:**
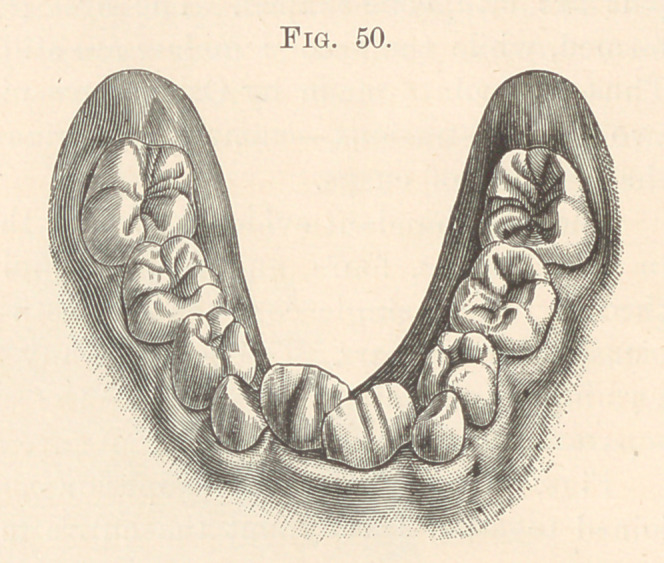


**Fig. 51. f21:**
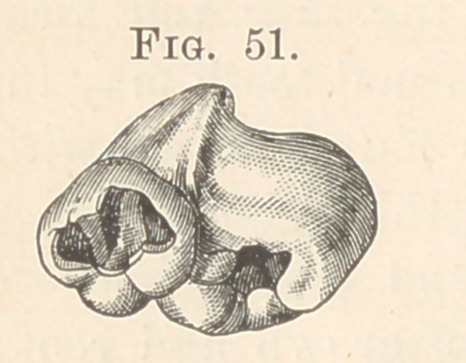


**Fig. 52. f22:**
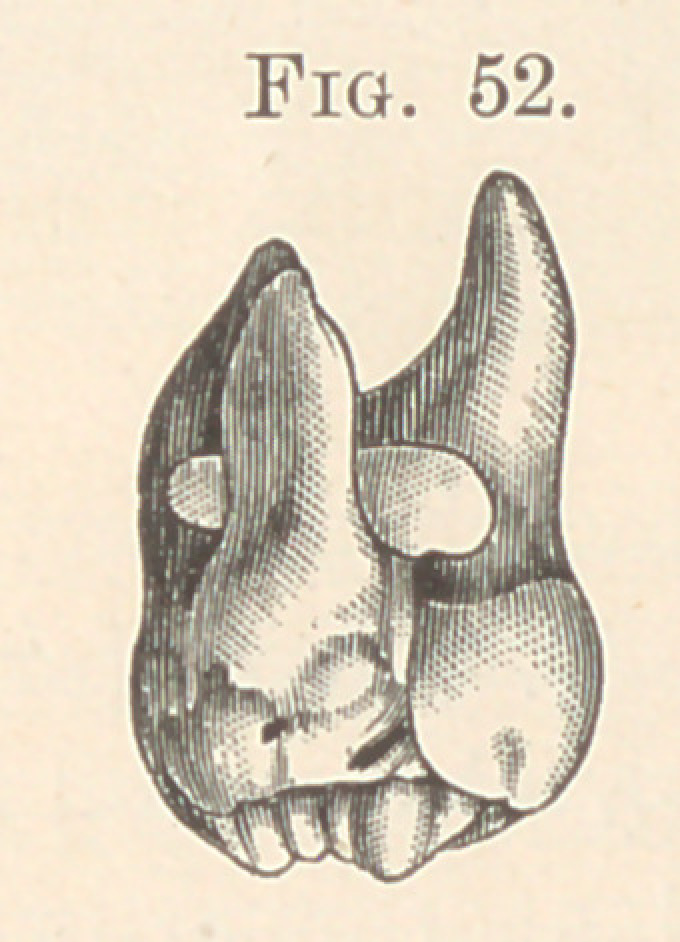


**Fig. 53. f23:**
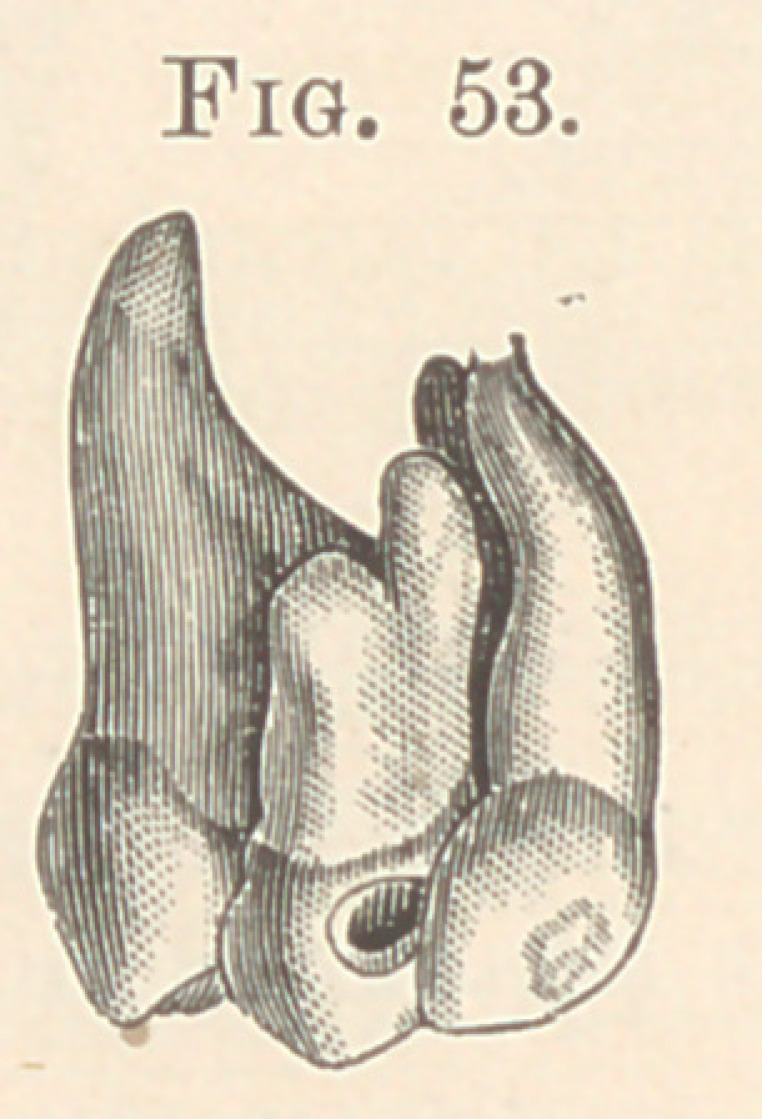


**Fig. 54. f24:**